# General dental practitioners' fees for root canal treatment, coronal restoration and follow‐on treatment in the adult population in Sweden: A 10‐year follow‐up of data from the Swedish Dental Register

**DOI:** 10.1002/cre2.826

**Published:** 2023-12-07

**Authors:** Emma Wigsten, Helena Fransson, Per‐Erik Isberg, EndoReCo, Victoria S. Dawson

**Affiliations:** ^1^ Department of Endodontology, Institute of Odontology, The Sahlgrenska Academy University of Gothenburg Gothenburg Sweden; ^2^ Department of Endodontics, Faculty of Odontology Malmö University Malmö Sweden; ^3^ Department of Statistics, Lund University School of Economics and Management Lund University Lund Sweden

**Keywords:** dental fees, endodontics, registry, tooth extraction

## Abstract

**Objectives:**

To analyze the accumulated fees connected with root filling, permanent coronal restoration and follow‐on treatment charged by Swedish dentists over a 10–11‐year follow‐up period. Furthermore, analyzing these fees with reference to the type of restoration, tooth group, and the root‐filled teeth which survived compared to those requiring extraction.

**Material and Methods:**

In 2009, the data register of the Swedish Social Insurance Agency recorded a total of 215,611 teeth as root‐filled. The accumulated fees for each tooth encompassed the following interventions: initial root filling, coronal restorations, and follow‐up treatments during the designated period. The outcomes were analyzed using descriptive and analytic statistics, including *t* tests and one‐way analysis of variance. The fees are presented in Euros (€1 = SEK 8.94).

**Results:**

The total accumulated fees for root fillings amounted to 72 million Euros: the mean fee per root filled tooth was €333.6. The total mean fee over a 10–11‐year period, comprising root canal treatment, coronal restorations, and any follow‐up treatments, was €923.4. Root‐filled teeth with indirect restorations presented a higher mean fee (€1 279.3) compared to those with direct restorations (€829.4) or those without specified restorations (€832.7; *p* < .001). Moreover, molars presented a significantly higher mean fee (€966.4) compared to premolars (€882.8) and anterior teeth (€891.3; *p* < .001). Lastly, the mean fee for extracted teeth was €1225.3, which was higher compared to those who survived the follow‐up period (€848.0; *p* < .001).

**Conclusions:**

Fees charged by general dental practitioners for root‐filled teeth accumulate over time, probably due to the need for further treatment of the tooth. The total mean fee was significantly higher for molars and root‐filled teeth with indirect restorations. However, an analysis of the total costs would require prospective clinical cost‐effectiveness studies.

## 1 INTRODUCTION

Health care is resource‐intensive and in many countries, costs continue to increase (Righolt et al., 2018; Schwendicke & Herbst, 2022). The global cost of oral disease, not only fees for dental care but also the cost incurred by loss of productivity, was estimated by the Global Burden of Disease Study in 2010 to be US $442 billion, with direct costs of $298 billion and indirect costs of $144 billion (Listl et al., 2015).

The estimated worldwide annual cost for treating oral diseases is $357 billion (Righolt et al., 2018). The most common condition, untreated caries in permanent teeth, afflicts 2.4 billion people (Kassebaum et al., 2015). If not treated, dental caries can lead to irreversible damage to the pulp: this is the main reason for endodontic treatment (Wigsten et al., 2019). Each year, more than 15 million root canal treatments are undertaken globally, with a higher prevalence in high‐income countries (Jones, 2016; Schwendicke & Herbst, 2022). For example, in Sweden, with a population of 10.5 million, dental care has a turnover of approximately €2.1 billion annually. Approximately 2% of the adult population undergoes root canal treatment annually (Fransson et al., 2016; Tandvårds‐ och läkemedelsförmånsverket [TLV], 2023).

After the tooth has been root‐filled and permanently restored, ideally no further treatment should be required. However, it is not uncommon for complications to arise in root‐filled teeth, necessitating further clinical intervention (Dawson et al., 2017). In some cases, extraction with possible replacement is considered instead, particularly when the outcome of the root canal treatment is unsatisfactory. Further intervention entails additional costs for the individual patient as well as for other stakeholders such as dentists, and any insurance companies or government benefit schemes which subsidize dental care (Schwendicke & Herbst, 2022; Wigsten et al., 2023; Zaror & Mariño, 2022).

There are remarkably limited studies assessing the long‐term cost associated with preserving a root‐filled tooth in the dentition. Likewise, there are a limited number of health economics studies in which endodontic treatment is compared with other relevant dental treatment (Schwendicke & Herbst, 2022; Wigsten et al., 2023). It is not unusual for a root‐filled tooth to require additional care, incurring further costs. As root canal treatment is a routine procedure in general dental practice, having information about the long‐term cumulative cost of maintenance is essential, and the absence of this information may complicate the decision‐making process (Azarpazhooh et al., 2016; Kvist & Hofmann, 2023; Schwendicke & Herbst, 2022). The follow‐up care is of importance not just to the patient and the dentist but also to third‐party providers. It is important to have data on the long‐term costs of different treatment options, especially when there is the possibility of replacement with, for example, implants (Iqbal & Kim, 2007). It is unclear whether the initial costly indirect restoration following the root filling, such as a dental technician fabricated crown, is the cheaper option in the longer term than the initial 5–6 years (Wigsten et al., 2018).

In this study, teeth recorded in the Swedish data register as root‐filled in 2009 were identified and followed through the register for a period of 10–11 years. The primary aim was to determine the overall accumulated fees charged by predominantly general dental practitioners for root fillings, subsequent coronal restorations, and any follow‐on treatment in Sweden. Further aims included comparing the fees for direct and indirect coronal restorations, different tooth groups, and analyzing the fees incurred for treatment of root‐filled teeth which survived compared to those requiring extraction

## 2 MATERIALS AND METHODS

### 2.1 Ethical considerations

The approval for this study was obtained from the Regional Ethical Committe in Lund, Sweden (Dnr 2011/800) and the Swedish Ethical Review Authority (Dnr 2020‐04856). The study was outlined according to the STROBE and PROBE checklist and statements (Nagendrababu et al., 2023; von Elm et al., 2008). This is a registry‐based study and none of the data contained information which could identify the participants.

### 2.2 The Swedish Dental Care Benefits Scheme

In Sweden, adults are responsible for covering the costs of their own dental care, but part of the fees may automatically be reimbursed by the government, calculated based on the government dental care subsidy (TLV, 2022a, 2023). The Dental Care Benefits Scheme is managed by the tax‐funded Swedish Social Insurance Agency (SSIA). This national financial support is generally available to most adult residents aged 20 years or older. The TLV (2022b) is responsible for deciding which treatments are eligible for reimbursement and establishing the calculation of scheduled fees (referred to as reference prices). The patient's share of the care costs and how much is to be compensated through SSIA are determined by the service provider's fee and the scheduled fee (TLV, 2022b).

In accordance with the subsidy system, the patient covers the entire cost for dental care up to €336. Treatment costs exceeding this are reimbursed by 50% of the reference prices up to €1 678 and 85% of costs more than this. The high‐cost protection system applies to all individuals for a period of 12 months. After this period expires, a new period may be started, again with full payment up to €336. SSIA's data register holds information about dental care provided, irrespective of whether the care was delivered privately or at a public dental clinic (TLV, 2022a, 2022b). In this study, the actual patient fee is investigated.

### 2.3 Root‐filled teeth in the SSIA's data register

All dental procedures included in the Dental Care Benefits Scheme have specific three‐digit codes. For example, root canal treatment is denoted by codes 501–504, corresponding to the number of root‐filled canals. All teeth registered as root‐filled (codes 501–504) in the SSIA's data register during 2009 were identified. These teeth were traced through the register for 10–11 years, concluding on December 31, 2019 or when the tooth was registered as extracted (codes 401–404).

The type of coronal restoration, specified within the 6 months following root filling, was classified for each tooth (Skupien et al., 2013). The categorization was sorted as “direct” restoration, which included a direct resin composite (codes 701–707), or “indirect” restoration, encompassing tooth‐supported crown, onlay, or inlay fabricated by a dental technician (codes 800, 801, 805–809, 921, 922). If no treatment code corresponding to either direct or indirect restoration was registered within the specified time period, the restoration was categorized as “unspecified”. In cases where both indirect and direct treatment codes were registered within this period, the tooth was classified as having undergone an indirect restoration.

By tracking each root‐filled tooth, all subsequent treatment codes and fees could be acquired from the register. The following procedures were tracked: additional coronal restoration, endodontic nonsurgical and surgical retreatment, and extraction (Wigsten et al., 2018). Table S1 presents the scheduled fees at baseline (2009) and at follow‐up (2019). The study excluded dental procedures that could not be referred to a specific tooth, such as the treatment of periodontal disease or preventive care. Conversion from Swedish Kronor (SEK) to Euros, was based on an exchange rate of €1 = SEK 8.94 (01/01/2012), which was the exchange rate utilized in the prior study (Wigsten et al., 2018).

Because a small proportion of Swedish dentists are specialists, for instance, only 0.6% are specialized in endodontics, the register essentially represents treatments performed by general dental practitioners (Socialstyrelsen, 2015).

### 2.4 Study population

In a previous study, fees were analyzed for almost a quarter of a million root canal treatments with accompanying root filling and further treatment of the tooth during the following 5–6 years (Wigsten et al., 2018). The study population encompassed 217,047 patients, with 49.8% women and 50.2% men, with a mean age of 55.1 years (standard deviation [SD] = 15.5, range: 20–102 years) (Fransson et al., 2016; Wigsten et al., 2018). Throughout the 5–6‐year follow‐up period, 25,228 teeth (10.2%) were registered as extracted. In this follow‐up study, only one root‐filled tooth per subject was included to minimize biases in the analyses. The first tooth with a treatment code 501–504 was included, resulting in the exclusion of 32,688 (13.2%) root‐filled teeth.

### 2.5 Statistical methods

Statistical analysis was conducted using IBM SPSS Statistics Version 29.0.0.0 (SPSS Inc.). The variables are presented as numbers with percentages, and the distribution is presented as mean values along with 95% confidence intervals, SDs, standard errors (SEs) and minimum and maximum. Comparisons between two groups were assessed using the *t* test (independent samples *t* test), and for comparisons involving three or more groups, a one‐way analysis of variance was used, followed by Tukeys' post hoc test. All statistical tests were analyzed at a significance level of 5% (*p* < .05).

## 3 RESULTS

This study comprises a total of 215,611 teeth that were registered as rootfilled in the SSIA's data register in 2009 and then tracked over a period of 10–11 years. The study encompasses an equal number of patients in its population. Molars predominated (*n* = 99,737, 46.3%), followed by premolars (*n* = 67,559, 31.3%) and anterior teeth (*n* = 48,315, 22.4%; Table 1).

**Table 1:** Total accumulated fees incurred for teeth registered in the Swedish Social Insurance Agency's data register as root‐filled in 2009, including a follow‐up period of 10–11 years.

					95% Confidence interval for mean				
Variable	Number (%)	Mean	SD	SE	Lower limit	Upper limit	Min	Max	*p*
Coronal restoration									<.001
Direct	128,461 (59.6)	829.4	572.1	1.60	826.3	832.5	0	54,152.1	
Indirect	44,732 (20.7)	1,279.3	573.2	2.7	1,274.0	1,284.6	0	16,569.4	
Unspecified	42,418 (19.7)	832.7	625.6	3.0	826.8	838.7	0	16,847.3	
Tooth group									<.001
Anterior	48,315 (22.4)	891.3	669.2	3.0	885.4	897.3	0	16,847.3	
Premolar	67,559 (31.3)	882.8	610.9	2.3	878.2	887.4	0	13,487.7	
Molar	99,737 (46.3)	966.4	577.8	1.8	962.9	970.0	0	54,152.1	
Tooth group, maxillary or mandibular									<.001
Maxillary anterior	34,270 (15.9)	955.6	703.2	3.8	948.2	963.1	0	16,847.3	
Mandibular anterior	14,045 (6.5)	734.5	547.1	4.6	725.4	743.5	0	8,173.9	
Maxillary premolar	39,805 (18.5)	923.1	633.7	3.2	916.9	929.4	0	13,487.7	
Mandibular premolar	27,754 (12.9)	825.0	571.7	3.4	818.2	831.7	0	8,364.1	
Maxillary molar	45,251 (21.0)	961.1	548.2	2.6	956.0	966.1	0	9,924.5	
Mandibular molar	54,486 (25.3)	970.9	601.2	2.6	965.8	975.9	0	54,152.1	
Total	215,611 (100.0)	923.4	611.0	1.3	920.8	926.0	0	54,152.1	

*Note*: The fees are presented in Euros. Total mean fees were calculated in relation to the type of coronal restoration provided within 6 months of the registered root filling (direct, indirect, or unspecified), tooth group, and by maxilla or mandible, respectively. The registered fees include all tooth‐specific treatment codes, such as the registered root filling in 2009, any further coronal restorations, and in some cases, additional endodontic treatment and extraction. Comparisons of total means between given groups were calculated by one‐way analysis of variance.

Abbreviations: Max, maximum; Min, minimum; *N*, number; SD, standard deviation; SE, standard error; Sig, significance.

### 3.1 Fees for root fillings in Sweden 2009

The total fees for the 215,611 root canal treatments, including root fillings, amounted to €71.9 million. The mean fee per root‐filled tooth was €333.6, €13.2 higher than the scheduled fee (€320.4), corresponding to a total difference of €2.84 million for the entire sample. The greatest mean difference between the actual charge and the scheduled fee was for teeth with four or more root canals (code: 504, *n* = 9371, €20.8).

### 3.2 Total accumulated fees for root‐filled teeth during long‐term follow‐up

The total fees for the 215,611 root‐filled teeth amounted to €199.1 million. The total mean fee for root filling and follow‐on interventions over the 10–11‐year follow‐up period is presented in Table 1. The total mean fee was €923.4 per root‐filled tooth (SD = €611.0). Most of the teeth were registered as directly restored (*n* = 128,461, 59.6%): the remainder were either indirectly restored (*n* = 44,732, 20.7%) or classified as unspecified (*n* = 42,418, 19.7%).

The total mean fees for root‐filled teeth with indirect restorations were significantly higher (€1279.3; Table 1) compared to directly restored teeth (€829.4) and those with unspecified restorations (€832.7; *p* < .001). The total mean difference between indirectly and directly restored teeth was €449.9 (SE = 3.2; *p* < .001), comparable with the difference between indirect and unspecified restorations: €446.6 (SE = 3.9; *p* < .001). There was no statistically significant difference between the direct and unspecified groups (SE = 3.3, *p* = .569).

With respect to total fees during the follow‐up period, statistically significant differences were observed among the tooth groups (Table 1; *p* < .001). Molars registered the highest total mean fee (€966.4), significantly higher than for premolars (€882.8, SE = 3.0; *p* < .001) and anterior teeth (€891.3, SE = 3.4; *p* < .001). The difference in fees between premolars and anterior teeth during the follow‐up period was also statistically significant (SE = 3.6; *p* = .050).

When dividing the tooth groups according to jaw, the highest total mean fee (€970.9, SD = 601.2; Table 1) was for mandibular molars, a statistically significant difference from maxillary premolars (€923.1, SE = 4.0; *p* < .001) and anterior teeth (€955.6, SE = 4.2; *p* = .004), mandibular premolars (€825.0, SE = 4.5; *p* < .001), and anterior teeth (€734.5, SE = 5.7; *p* < .001). However, the difference between mandibular and maxillary molars was not significant (€961.1, SE = 3.6; *p* = .113).

### 3.3 Total fees for root‐filled teeth which survived compared with those which were extracted

Over the 10–11‐year follow‐up period, 43,087 root‐filled teeth (20.0%) were registered as having been extracted in the SSIA's data register. Overall, the total mean fees for root‐filled teeth that underwent extraction during the follow‐up period were significantly higher (€1225.3, SD = 886.7) than for those which were successfully retained (€848.0, SD = 491.6; *p* < .001).

## 4 DISCUSSION

The study analyzed fees charged by general dental practitioners for 215,611 teeth registered as root‐filled in the SSIA's data register in 2009 (Fransson et al., 2016). The mean fee for root canal treatment and root filling was approximately €333.6. The total accumulated mean fee for root canal treatment, subsequent root filling, coronal restoration, and any follow‐on treatment over the following 10–11 years was €923.4. Indirectly restored teeth presented a higher mean fee than those directly restored. The highest total mean fees were registered for molars. Finally, total mean fees were greater for extracted teeth compared to those retained in the dentition. The data retrieved are from the Swedish adult population (>20 years). The original study included 248,299 teeth registered as root‐filled in the data register of SSIA in 2009 (Dawson et al., 2017; Fransson et al., 2016; Wigsten et al., 2018). In the follow‐up study, only one tooth per individual was included to minimize biases, which means that 32,688 (13.2%) root‐filled teeth were excluded.

Registry‐based studies have the advantage of enabling the analysis of larger populations. The register thus provides an important overview of root canal treatment performed in general dental practice, including further interventions if required. The data cover patients treated in both the private and public sectors. However, the register does not offer clinical documentation such as dental records or radiographs, which precludes more detailed study. Controlled clinical trials would be required to investigate more specific questions about endodontic treatment and its costs (Schwendicke & Herbst, 2022; Statens Beredning för Medicinsk Utvärdering [SBU], 2010; Zaror & Mariño, 2022). The results are based on the dentists' registration with the SSIA and therefore the internal validity is considered good. External validity could be considered good, provided that the organization of dental services is similar to that described here. Nevertheless, this study confirms that based on the evidence of the fees charged, teeth which are treated by general dental practitioners can require further treatment in years to come, including extraction in some cases. There are few studies that have analyzed the fees for root canal treatment and the resources required to maintain the root‐filled tooth as a functional unit over time (Wigsten et al., 2023). Under ideal conditions, once the tooth has been root‐filled and permanently restored, no further treatment should be required. One study by Azarpazhooh et al. (2016) studied patients' opinions when choosing treatment for a tooth diagnosed with apical periodontitis, and the costs of subsequent care were highly valued. These costs should be discussed already at the start of treatment to prolong the survival of root‐filled teeth.

During the first 5–6 years, a total mean fee of €717 per tooth was incurred. This included the initial root filling, coronal restoration, any subsequent endodontic interventions and possibly extraction (Dawson et al., 2017; Fransson et al., 2016; Wigsten et al., 2018). Three percent (*n* = 7004) required additional endodontic surgical or nonsurgical intervention (Dawson et al., 2017) and one‐tenth of the teeth were registered as extracted (Fransson et al., 2016). Over the following 5 years, the total fee for all additional interventions increased by a mean of 30 percent per tooth (from €717 to €923.4). Hypothetically, this could imply that the total fee of €199 million represents an increase of over 40%. These fees represent the cost of treatment of the root‐filled teeth over a decade. Only tooth‐specific codes are included, thus the actual total fee may be somewhat underestimated, as preventive care, periodontal treatment or exploratory surgery are nontooth‐specific codes and therefore not included. Over the 10–11‐year follow‐up, approximately 20% of the root‐filled teeth were registered as having been extracted.

Most of the root‐filled teeth were registered as having undergone direct restoration (59.6%) within 6 months following the completion of root canal treatment (Table 1). However, a notable proportion of the teeth (19.7%) did not have registrations of either indirect or direct coronal restoration. The specific reasons for this are unknown, but one presumable explanation could be that some teeth were permanently restored beyond the 6‐months period following root filling.

During the 5 to 6 years of follow‐up, one‐third of the teeth restored with a direct restoration required at least one additional direct restoration to replace or repair the restoration, compared with 6% of the teeth with indirect restorations (*p* < .001; Dawson et al., 2017). Hypothetically, despite the initial higher fee for an indirect restoration, in a longer time perspective the mean fee may be lower than for directly restored teeth. However, this was not confirmed in the present study, in which a significantly higher total mean fee, almost twofold, was charged for indirectly restored root‐filled teeth compared to teeth registered as directly restored or unspecified. The results from the previous study indicate that the situation remains the same; the total mean fee continued to be significantly higher for root‐filled teeth with indirect restorations compared to teeth with direct restorations, even after 5–6 years (Wigsten et al., 2018). The highest mean increase, from €585.0 to €832.7, an increase of 42% was in those registered with unspecified restorations. The teeth restored with indirect restorations showed an increase of 15% (€1105.0–€1279.3).

The molars continued to present the highest total mean fee and maximum fee. In Sweden, root canal treatment is charged based on the number of root‐filled canals, resulting in a higher initial fee for molars, as they commonly have three or more root canals (Table S1).

Despite that root canal treatment of molars is a frequent procedure in Scandinavian general dental practice (Bjørndal et al., 2006; Försäkringskassan, 2023; Fransson et al., 2016), the outcomes seem to be problematic. Some studies not only indicate the poor technical quality of the root fillings but also the absence of periapical healing (Eckerbom et al., 2007; Frisk et al., 2008; Laukkanen et al., 2021; Silnovic et al., 2023). The survival rates for root‐filled molar teeth are generally lower than for other tooth groups treated in general practice (Fransson et al., 2016, 2021; Göransson et al., 2021; Kebke et al., 2021). There could be several contributing factors to this concern. Root canal treatment of molars is often more complex and technically more challenging compared to other tooth groups (Peters, 2016). Molars often have multiple roots and a more complicated root canal anatomy, and their posterior position in the dental arch can make access challenging. Moreover, not only is the technical aspect demanding, but molar root canal treatment often requires more resources (Peters, 2016). However, tooth extraction is not solely performed for endodontic reasons; other common causes include fractures and dental caries (Kebke et al., 2021).

The mean fee was lower for the root‐filled teeth retained for a decade than for those which were subsequently extracted (€848.0 and €1225.3, respectively; *p* < .001). The difference might be attributable to fees for further necessary treatment of the tooth, such as an additional fee for extraction. In 2009, the extraction fee varied between €84 and €303, contingent upon the treatment's complexity. The annual mean loss of root‐filled teeth continued at approximately 2 percent, aligning with findings from other Scandinavian studies (Fransson et al., 2016; Göransson et al., 2021; Kebke et al., 2021).

In this study, the focus was not on the costs; rather, it centered on the fees charged by general dental practitioners. To study the costs comprehensively, additional factors should be considered, such as the number of appointments, treatment duration, material expenses, patients' absence from work, travel to and from the dental clinic, and so forth. To study the cost‐effectiveness, in addition to the costs of treatment, an appropriate outcome measure should also be selected: this could reasonably be quality of life (Drummond et al., 2015; Schwendicke & Herbst, 2022; Wigsten et al., 2023; Zaror & Mariño, 2022). Cost‐effectiveness studies are necessary to ensure that the limited resources available provide the greatest possible health gains. The few studies to date have analyzed cost‐effectiveness primarily through so‐called modeling: there are few studies based on empirical data (Kim & Solomon, 2011; Pennington et al., 2009; Schwendicke & Göstemeyer, 2016; Schwendicke & Herbst, 2022; Schwendicke & Stolpe, 2015; Wigsten et al., 2023). Clinical follow‐up studies are warranted. While the main focus should be on fees and costs, the cost‐effectiveness should also be compared with that of other relevant treatments (Schwendicke & Herbst, 2022; Wigsten et al., 2023). The next step could be a randomized study comparing root canal treatment and permanent restoration with extraction and replacement using implants or other prosthetic constructions. However, there may be ethical barriers to such a study.

## 5 CONCLUSION

Fees charged by general dental practitioners for root‐filled teeth accumulated over time, primarily because of the need for further treatment of the tooth. The total mean fee was significantly higher for molars and root‐filled teeth with indirect restoration compared to those with direct restoration. To analyze the total costs, however, would require prospective clinical cost‐effectiveness studies.

## AUTHOR CONTRIBUTIONS

Emma Wigsten contributed to conception, design, data acquisition, analysis, and interpretation, drafted the manuscript. Helena Fransson, Victoria S. Dawson contributed to conception, design, data acquisition, analysis, and interpretation, critically revised the manuscript. Per‐Erik Isberg contributed to data acquisition and analysis, critically revised the manuscript. All authors gave final approval and agreed to be accountable for all aspects of the work. The collaborators in EndoReCo have critically revised the manuscript.

## ENDODONTIC RESEARCH COLLABORATION

The researchers within the Endodontic Research Collaboration in Scandinavia contributed to this study. Collaborators: Lars Bjørndal, Victoria S. Dawson, Helena Fransson, Fredrik Frisk, Peter Jonasson, Thomas Kvist, Merete Markvart, Maria Pigg, and Emma Wigsten.

## CONFLICT OF INTEREST STATEMENT

The authors declare no conflict of interest.

## ETHICS STATEMENT

This study was approved by the Regional Ethical Committee in Lund, Sweden (Dnr 2011/800) and the Swedish Ethical Review Authority (Dnr 2020‐04856). The research has been conducted in full accordance with ethical principles, including the World Medical Association Declaration of Helsinki (version 2008) and the requirements of Swedish law, under which the research has been conducted. The material was retrieved from Swedish Social Insurance Agency's data register. The data did not contain any information that could identify the participants.

## Supporting information

Supporting information.

Supporting information.

Supporting information.

Supporting information.

Supporting information.

Supporting information.

## Data Availability

The data that support the findings of this study are available from the corresponding author, Emma Wigsten, upon reasonable request.
