# Supplementary material for: General dental practitioners' fees for root canal treatment, coronal restoration and follow‐on treatment in the adult population in Sweden: A 10‐year follow‐up of data from the Swedish Dental Register
Source: Clin Exp Dent Res. 2023 Dec 7;10(1):e826. doi: 10.1002/cre2.826 (PMC10860445; doi:10.1002/cre2.826)
Supplement: Supplementary file 1 — Supporting information. [file CRE2-10-e826-s002.docx]

**Supplemental table.** Tooth-specific treatment items and scheduled fees. All fees are presented in Euro (€1 = SEK 8.94; 01/01/2012).

| **Treatment code** | | **Scheduled fees at baseline** (01/01/2009) | **Scheduled fees**  **at follow-up** (31/12/2019) |
| --- | --- | --- | --- |
| **Endodontic treatment** | |  |  |
| Root canal treatment of: | |  |  |
|  | 1 root canal (501) | 225 | 380 |
|  | 2 root canals (502) | 286 | 458 |
|  | 3 root canals (503) | 393 | 574 |
|  | 4 root canals (504) | 464 | 626 |
|  | Additional fee for complicated root canal localization (522) | 72 | 90 |
|  | Root-end surgery (541) | 315 | 409 |
| **Direct restorations (*i.e.* composite)** | |  |  |
| Restoration of: | |  |  |
|  | *Incisors or canines* |  |  |
|  | 1 surface (701) | 57 | 68 |
|  | 2 surfaces (702) | 89 | 107 |
|  | 3 or more surfaces (703) | 106 | 128 |
|  | *Premolars or molars* |  |  |
|  | 1 surface (704) | 72 | 87 |
|  | 2 surfaces (705) | 107 | 128 |
|  | 3 or more surfaces (706) | 140 | 169 |
|  | Full cover crown (707) | 162 | 193 |
| **Indirect restorations (*i.e.* fabricated by a dental technician)** | | |  |
|  | Tooth-supported crown/onlay/inlay (800)^1^ | 608 | 644 |
|  | Tooth-supported crown/onlay/inlay (801) | 496 | 500 |
|  | Resin-bonded prosthesis, per unit (805) | 168 | 210 |
|  | Radicular anchorage for removable dental prosthesis (806) | 359 | 383 |
|  | Temporary crown or pontic, per unit (with metallic framework) (807) | 213 | 275 |
|  | Inner crown for telescopic or conus removable dental prosthesis, per unit (808) | 326 | 376 |
|  | Long-term temporary crown or pontic, per unit (809)^2^ | 116 | 117 |
|  | Replacement procedure, indirect restoration instead of direct filling (incisor or canine, 921)^2^ | 113 | 128 |
|  | Replacement procedure, indirect restoration instead of direct filling (premolar or molar, 922)^2^ | 148 | 169 |
| **Extractions** | |  |  |
|  | Uncomplicated extraction (401) | 84 | 115 |
|  | Complicated extraction (402) | 159 | 189 |
|  | Additional extraction in the same area (403) | 16 | 21 |
|  | Extraction requiring surgery (404) | 239 | 349 |

The fees of the treatment items are dated onward from 01/09/2014^1^ and 01/07/2010^2^
