# Supplementary material for: General dental practitioners' fees for root canal treatment, coronal restoration and follow‐on treatment in the adult population in Sweden: A 10‐year follow‐up of data from the Swedish Dental Register
Source: Clin Exp Dent Res. 2023 Dec 7;10(1):e826. doi: 10.1002/cre2.826 (PMC10860445; doi:10.1002/cre2.826)
Supplement: Supplementary file 2 — Supporting information. [file CRE2-10-e826-s003.pdf]

REGIONALA ETIKPRÖVNINGSNÄMNDEN
EPN
LUND
Box 133, 221 00 Lund
Avdelning 3
046-222 46 16

PROTOKOLL VETENSKAPLIG SEKRETERARE

2012/5
2012-02-28

Närvarande Ulf Görman, vetenskaplig sekreterare

Dnr 2011/800

Forskningshuvudman
Malmö Högskola

Forskare som genomför projektet (kontaktperson)
Kerstin Petersson

Projekttitel
Tandöverlevnad efter rotbehandling i Sverige.
Projektnummer/identitet: Tooth Survival Version nummer: 2011-12-19.

Beslut

Enligt delegation beslutar vetenskaplig sekreterare att komplettering inkommen 2012-02-21 , godkänns med följande villkor:

- Kodade data utgör indirekt identifierbara personuppgifter i personuppgiftslagens mening. Skyddsprinciperna i lagen är därför tillämpliga och ska beaktas under all behandling av data i projektet. All bearbetning och förvaring av data ska äga rum på dator eller annat medium utan anslutning till internet.

Beträffande hur man överklagar, se bilaga 1

Vid protokollet

Ann-Marie Kellner
Administrativ sekreterare

Justeras

Ulf Görman
Vetenskaplig
sekreterare

Exp till: Kerstin Petersson
