# Supplementary material for: General dental practitioners' fees for root canal treatment, coronal restoration and follow‐on treatment in the adult population in Sweden: A 10‐year follow‐up of data from the Swedish Dental Register
Source: Clin Exp Dent Res. 2023 Dec 7;10(1):e826. doi: 10.1002/cre2.826 (PMC10860445; doi:10.1002/cre2.826)
Supplement: Supplementary file 3 — Supporting information. [file CRE2-10-e826-s004.pdf]

2020-04856 Beslut godkänd.pdf

Signers:

Name	*Method*	*Date*
ANNA BILLING	BANKID	2020-10-06 15:05 GMT+2

This document package contains:

- Front page (this page)
- The original document(s)
- The electronic signatures. These are not visible in the document, but are electronically integrated.

This file is sealed with a digital signature. The seal is a guarantee for the authenticity of the document.

Document ID:
49FFA2B0BB064FA7B774BF0B3659DFCE

THE SIGNED DOCUMENT FOLLOWS ON THE NEXT PAGE >

Dnr 2020-04856

Lund avdelning övrig

BESLUT
2020-10-06

Sökande forskningshuvudman
Malmö högskola

Forskare som genomför projektet
Kerstin Petersson

Projekttitel
Tandöverlevnad efter rotbehandling i Sverige

Aktuell ändring
Ansökan om ändring inkommen 2020-09-14.

Grundansökan godkänd 2012-02-28 av Regionala etikprövningsnämnden i Lund med diarienummer 2011/800.

Etikprövningsmyndigheten beslutar enligt nedan.

BESLUT

Etikprövningsmyndigheten godkänner den forskning som anges i ansökan om ändring.

På Etikprövningsmyndighetens vägnar

Anna Billing
Ordförande

Beslutet har fattats efter föredragning av vetenskaplig sekreterare Staffan Karlsson.

Beslutet sänds till
Ansvarig forskare: Kerstin Petersson

This file is sealed with a digital signature. The seal is a guarantee for the authenticity of the document.
Document ID: 49FFA2B0BB064FA7B774BF0B3659DFCE
