# Supplementary material for: General dental practitioners' fees for root canal treatment, coronal restoration and follow‐on treatment in the adult population in Sweden: A 10‐year follow‐up of data from the Swedish Dental Register
Source: Clin Exp Dent Res. 2023 Dec 7;10(1):e826. doi: 10.1002/cre2.826 (PMC10860445; doi:10.1002/cre2.826)
Supplement: Supplementary file 4 — Supporting information. [file CRE2-10-e826-s005.docx]

| **Original (Swedish)** | **Translation** |
| --- | --- |
| Regionala etikprövningsnämnden (EPN)  Box 133, 221 00 Lund  Avdelning 3  046-222 46 16 | Regional Ethical Committee (EPN)  Adress: Box 133, 221 00 Lund  Department 3  Phone number: 046-222 46 16 |
| **Protokoll vetenskaplig sekreterare**  2012/5  2012-02-28 | **Protocol scientific secretary**  2012/5  28/02/2012 |
| **Närvarande**  Ulf Görman, vetenskaplig sekreterare | **Attendant**  Ulf Görman, scientific secretary |
| Dnr 2011/800 | Dnr 2011/800 |
| Forskningshuvudman  Malmö Högskola | Entity responsible for research Malmö University |
| Forskare som genomför projektet (kontaktperson)  Kerstin Petersson | Principal investigator (contact person) Kerstin Petersson |
| Projekttitel  Tandöverlevnad efter rotbehandling i Sverige.  Projektnummer/identitet: Tooth Survival Version nummer: 2011-12-19 | Title of the project  Tooth survival after root canal treatment in Sweden.  Number of the project/identity: Tooth Survival Version number: 2011-12-19 |
| **Beslut** | **Decision** |
| Enligt delegation beslutar vetenskaplig sekreterare att komplettering inkommen 2012-02-21, godkänns med följande villkor:   - Kodade data utgör indirekt identifierbara personuppgifter i personuppgiftslagens mening. Skyddsprinciperna i lagen är därför tillämpliga och ska beaktas under all behandling av data i projektet. All bearbetning och förvaring av data ska äga rum på dator eller annat medium utan anslutning till internet. | According to delegation, the scientific secretary decides that the addendum received on 21/02/2012 is approved with the following conditions:   - encoded data constitutes indirectly identifiable personal data within the meaning of the Personal Data Act. The protection principles in the law are therefore applicable and must be taken into account in all processing of data in the project. All processing and storage of data must take place on a computer or other medium without connection to the Internet. |
| Beträffande hur man överklagar, se bilaga 1 | Regarding how to appeal, see Appendix 1 |
| Vid protokollet  Ann-Marie Kellner  Administrativ sekreterare | At the protocol  Ann-Marie Kellner  Administrative secretary |
| Justeras  Ulf Görman  Vetenskaplig sekreterare | Adjusted by  Ulf Görman  Scientific secretary |
| Exp till: Kerstin Petersson | Sent to: Kerstin Petersson |
