# Supplementary material for: General dental practitioners' fees for root canal treatment, coronal restoration and follow‐on treatment in the adult population in Sweden: A 10‐year follow‐up of data from the Swedish Dental Register
Source: Clin Exp Dent Res. 2023 Dec 7;10(1):e826. doi: 10.1002/cre2.826 (PMC10860445; doi:10.1002/cre2.826)
Supplement: Supplementary file 5 — Supporting information. [file CRE2-10-e826-s006.docx]

| **Original (Swedish)** | **Translation** |
| --- | --- |
| 2020-04856 Beslut godkänd.pdf | 2020-04856 Decision approved.pdf |
| Signers:  Name  Anna Billing | Signers:  Name  Anna Billing |
| Dnr 2020-04856 | Dnr 2020-04856 |
| Lund avdelning övrig | Lund department other |
| **Beslut**  2020-10-06 | **Decision**  06/10/2020 |
| **Sökande forskningshuvudman**  Malmö högskola | Entity responsible for research Malmö University |
| **Forskare som genomför projektet**  Kerstin Petersson | Principal investigator Kerstin Petersson |
| **Projekttitel**  Tandöverlevnad efter rotbehandling i Sverige | **Title of the project**  Tooth survival after root canal treatment in Sweden |
| **Aktuell ändring**  Ansökan om ändring inkommen 2020-09-14. | **Amendment**  Application for amendment received 14/09/2020. |
| Grundansökan godkänd 2012-02-28 av Regionala etikprövningsnämnden i Lund med diarienummer 2011/800. | The original application was approved on 28/02/2012 by the Regional Ethical Committee in Lund with registration number (Dnr) 2011/800. |
| Etikprövningsmyndigheten beslutar enligt nedan. | The Swedish Ethical Review Authority decides as below. |
| **Beslut** | **Decision** |
| Etikprövningsmyndigheten godkänner den forskning som anges i ansökan om ändring. | The Swedish Ethical Review Authority approves the research specified in the application for amendment. |
| På Etikprövningsmyndighetens vägnar | On behalf of the Swedish Ethical Review Authority |
| Anna Billing  Ordförande | Anna Billing  Chairman |
| Beslutet har fattats efter föredragning av vetenskaplig sekreterare  Staffan Karlsson. | The decision has been taken following a presentation by the scientific secretary  Staffan Karlsson. |
| **Beslutet sänds till**  Ansvarig forskare: Kerstin Petersson | The decision is sent to:  Principal investigator: Kerstin Petersson |
